# Differences in gait apraxia due to reduced regional cerebral blood flow in the supplementary motor area in corticobasal syndrome: a report of two cases

**DOI:** 10.1186/s12883-025-04332-z

**Published:** 2025-07-29

**Authors:** Kota Igari, Motoki Fujimaki, Mera Mai, Moe Sakuma, Shinji Saiki

**Affiliations:** 1 Department of Neurology, Faculty of Medicine, University of Tsukuba, 1-1-1 Tennodai, Tsukuba, Ibaraki 305-8575 Japan; 2 Center for Medical Education and Training, University of Tsukuba Hospital, Tsukuba, Ibaraki Japan

**Keywords:** Gait apraxia, Corticobasal syndrome, Supplementary motor area, Regional cerebral blood flow, Freezing gait, Neurodegeneration

## Abstract

**Background:**

Gait apraxia, characterized by difficulties initiating and coordinating walking despite preserved conceptual movement abilities, is a distinct entity from lower limb apraxia. Although gait apraxia has been associated with dysfunction of the frontal lobe, particularly the supplementary motor area (SMA), the specific associated somatotopic organization phenotype remains poorly understood. Corticobasal syndrome (CBS), a clinical phenotype of corticobasal degeneration, commonly presents with upper limb apraxia, while lower limb or gait apraxia has rarely been reported. Herein, we describe two rare cases of CBS presenting with gait apraxia shown to be caused by SMA dysfunction, based on regional cerebral blood flow (rCBF) reduction on single-photon emission computed tomography (SPECT).

**Case presentation:**

Case 1 was of an 82-year-old man who exhibited right-sided apraxic gait with freezing and shuffling patterns, along with SMA hypoperfusion in both the dorsal and pre-SMA regions. Neurological examination revealed mild rigidity, right-sided Babinski sign, and clumsiness in mimicking leg movements. Gait patterns were inconsistent and unresponsive to levodopa treatment or sensory cues. Case 2 was of an 80-year-old man who demonstrated a peculiar gait characterized by exaggerated right leg movements and everted ankle positioning. Hypoperfusion was localized to the left dorsal SMA. Examination findings included rigidity and impaired hand weight perception. Sensory tricks and levodopa provided no benefit.

**Conclusion:**

These cases highlight the role of SMA dysfunction in the pathogenesis of gait apraxia. Variations in rCBF reduction correlated with distinct gait patterns. For example, the freezing gait shown in Case 1 likely resulted from pre-SMA impairment, which is critical for movement initiation, while the exaggerated leg movements in Case 2 reflected dorsal SMA dysfunction, involved in motor execution. These results indicate that gait apraxia, which is often underdiagnosed, should be recognized as a potential early indicator of CBS. Further, these cases suggest that SMA dysfunction, identified through SPECT imaging, underlies the distinct gait patterns seen in CBS patients with apraxic gait. Recognizing these symptoms, even in the absence of weakness or limb apraxia, may aid in early CBS diagnosis and improve clinical management.

**Supplementary Information:**

The online version contains supplementary material available at 10.1186/s12883-025-04332-z.

## Background

Gait apraxia, although not formally classified as “apraxia,” is characterized by difficulties in initiating walking, episodes of freezing, short shuffling steps, impaired balance, and challenges in postural transitions, after excluding other potential causes [1]. In addition, gait apraxia is distinguished from lower limb apraxia based on the ability of the patient to perform conceptual movements [2]. Although the symptoms and the functional localization underlying gait apraxia have been discussed extensively with some discrepancies, it is widely acknowledged that frontal impairment—particularly in the supplementary motor area (SMA) and premotor area—is a key factor in its development [1–3]. However, the discussion about the association between the functional organization of SMA and gait apraxia is insufficient in previous reports.

Corticobasal degeneration (CBD) exhibits various clinical phenotypes, including frontal behavioral-spatial syndrome, nonfluent/agrammatic variant of primary progressive aphasia, progressive supranuclear palsy syndrome, and corticobasal syndrome (CBS) [4], with CBS being the most common. CBS is a progressive neurodegenerative disorder characterized by akinetic rigidity and asymmetric limb apraxia, involving cortical and basal ganglia dysfunction. Although several diagnosis criteria for CBS have been proposed, they all share the common feature of including limb apraxia as a key clinical feature. While upper limb apraxia onset is typical for CBS, lower limb apraxia onset has also been reported in several cases. In addition, gait apraxia, although not typically emphasized, is likely underdiagnosed in these patients.

Herein, we present two extremely rare cases of gait apraxia, likely related to SMA dysfunction in patients with CBS. By comparing the two cases with slightly different ambulation patterns, we discuss the somatotopy and functional localization of the SMA as they are related to gait [5–7]. Furthermore, we emphasize the importance of identifying gait apraxia for aiding early diagnosis.

## Case presentations

### Case 1

**Fig. 1:**
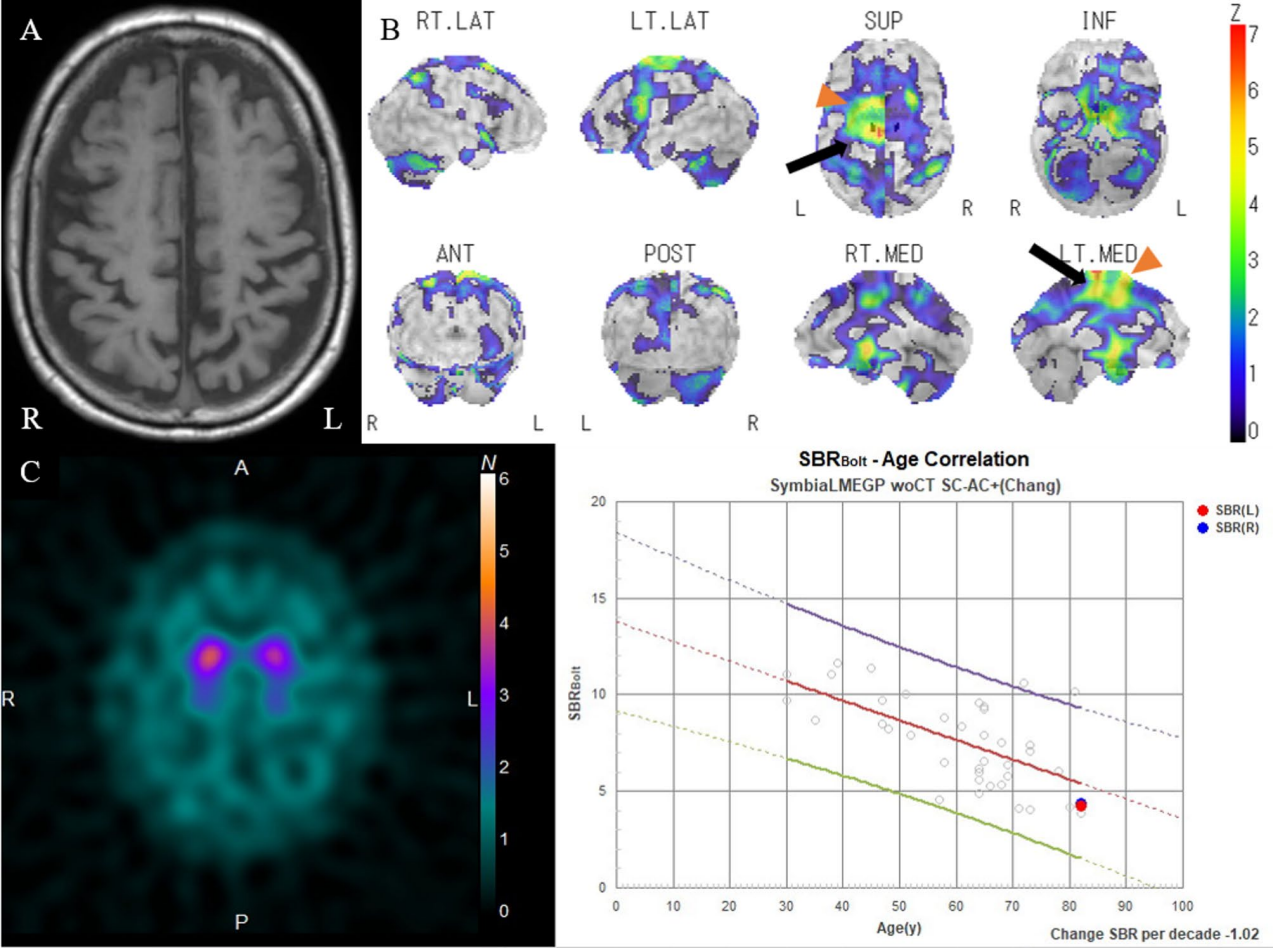
Magnetic resonance images (MRIs) and three-dimensional stereotactic surface projection (3D-SSP) analysis images of 123I-IMP SPECT of case 1. Axial T1-weighted image shows suspected generalized atrophy, particularly in the parietal lobes (**A**). 3D-SSP analysis images of 123I-IMP SPECT indicate the reduction of regional cerebral blood flow in the left pre-SMA (arrowhead) and left dorsal SMA (arrow), with no reduction in basal ganglia. Color-coding represents the statistical significance (Z-score) of the decrease in regional cerebral blood flow (**B**). Dopamine transporter scan shows visually mild hypo-accumulation, with specific binding ratio values of 4.39 and 4.22 on the right and left sides, respectively (**C**)

Case 1 was of an 82-year-old man who presented with a 2-year history of right lower limb mobility impairment, followed by speech difficulty 1 year later. Neurological findings included diminished verbal fluency, dyscalculia, mild lower limb rigidity, and a right-sided Babinski sign. There were no clinical signs indicative of bradykinesia, dystonia, or myoclonus. He exhibited mild clumsiness in drawing circles and mimicking kicking a ball (Video 1). Ideational and ideomotor apraxia, alien hand syndrome, cortical sensory loss, muscle weakness, sensory loss, and cerebellar or sensory ataxia were absent. During walking, he pivoted on his left foot, with impaired right foot movement, resembling a “freezing gait” or “shuffling gait” (Video 1). Moreover, his right ankle remained everted while walking. Unlike typical gait freezing, his apraxic gait displayed various disorganized patterns with each step and was unresponsive to sensory tricks. Moreover, the gait pattern did not reveal any sustained muscle contractions suggestive of dystonia. He was also nonresponsive to levodopa treatment. His cerebrospinal fluid (CSF) total tau (t-tau) protein and phosphorylated tau (p-tau) protein concentrations were 265 and 40.9 pg/mL, respectively. Brain magnetic resonance imaging (MRI) revealed suspected generalized atrophy, particularly in the parietal lobes (Fig. 1A). Moreover, 123I-IMP SPECT imaging revealed reduced regional cerebral blood flow (rCBF) in the left pre-SMA and left dorsal SMA (Fig. 1B). Although a dopamine transporter scan indicated visually mild hypo-accumulation, the specific binding ratios were within the normal range (right side, 4.39; left side, 4.22) (Fig. 1C). Therefore, the patient was diagnosed with CBS according to the Revised Cambridge Criteria [8].

### Case 2

**Fig. 2:**
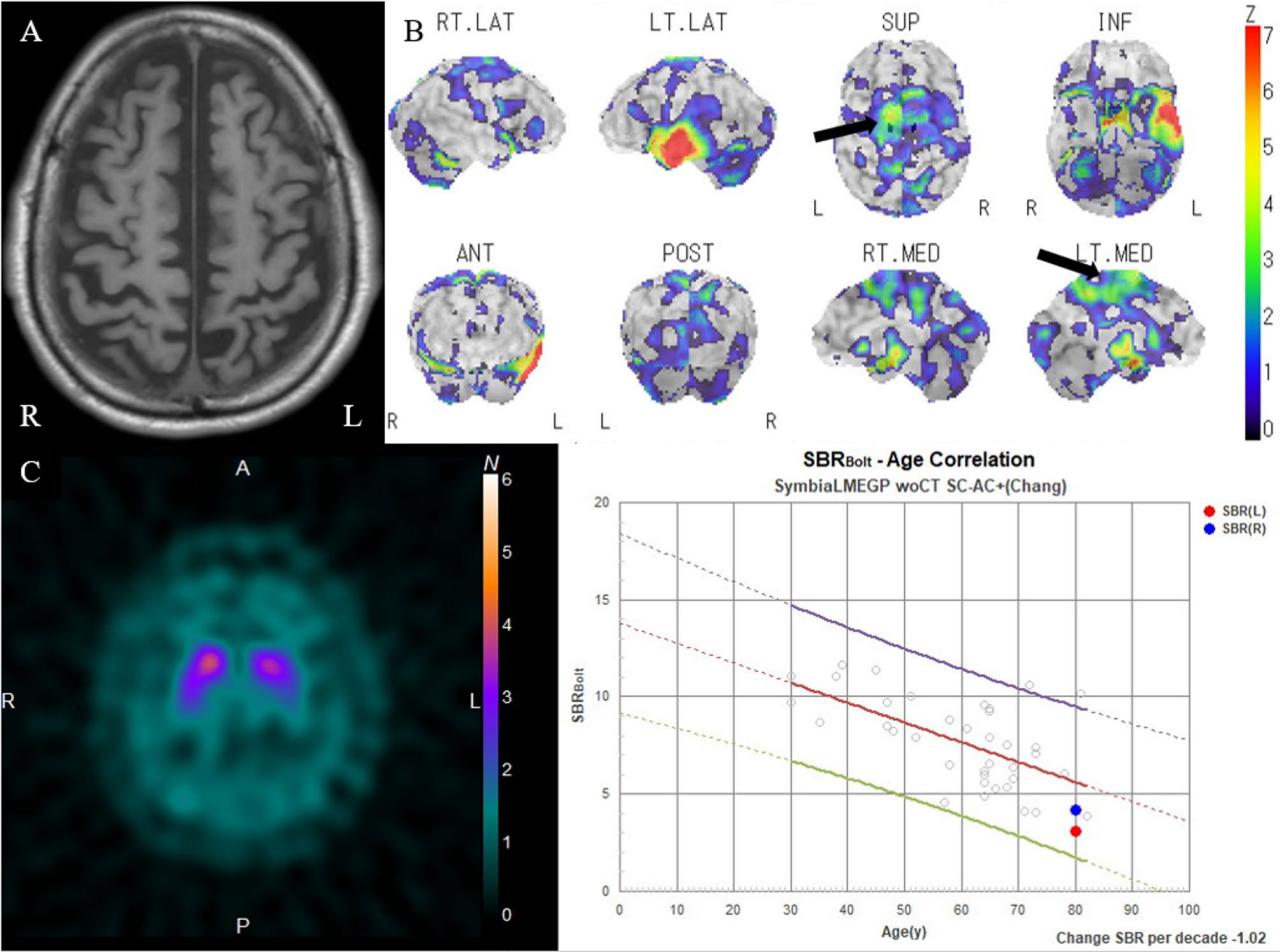
Magnetic resonance images (MRIs) and three-dimensional stereotactic surface projection (3D-SSP) analysis images of 123I-IMP SPECT of case 2. Axial T1-weighted image shows atrophy of the left temporal lobe due to prior cerebral infarction and generalized atrophy, particularly in the parietal lobes (**A**). 3D-SSP analysis images of 123I-IMP SPECT indicate a reduction in regional cerebral blood flow in the left dorsal SMA (arrow), without basal ganglia reduction. Color-coding represents the statistical significance (Z-score) of the decrease in regional cerebral blood flow (**B**). A dopamine transporter scan demonstrates visually mild left-dominant hypo-accumulation, with specific binding ratio values of 4.17 and 3.06 on the right and left sides, respectively (**C**)

Case 2 was of an 80-year-old man who presented with a 1-year history of right lower limb mobility impairment, which worsened to hindrance in walking 5 months later. Neurological findings included a Frontal Assessment Battery score of 9/18, impaired hand weight perception, mild upper and lower limb rigidity, and a right-sided Babinski sign. There were no clinical signs indicating bradykinesia, dystonia, or myoclonus. He experienced slight difficulty in kicking a ball, with no issues in drawing circles and crosses (Video 2). Ideational and ideomotor apraxia, alien hand syndrome, muscle weakness, and sensory loss were absent. He exhibited a peculiar gait pattern, lifting the right leg high, swinging it outward, and maintaining the right ankle in an everted position (Video 2). Sensory tricks did not alleviate symptoms, and he was nonresponsive to levodopa treatment. The gait pattern showed no sustained muscle contractions. His CSF t-tau and p-tau protein concentrations were 220 and 34.7 pg/mL, respectively. Brain MRI indicated suspected generalized atrophy, particularly in the parietal lobes (Fig. 2A). SPECT revealed reduced rCBF in the left dorsal SMAs (Fig. 2B). A dopamine transporter scan indicated visually mild hypo-accumulation, and the specific binding ratios were within the normal range (right side, 4.17; left side, 3.06) (Fig. 2C). Therefore, the patient was diagnosed with CBS according to the Revised Cambridge Criteria [8].

## Discussion

These two cases highlight the following points: First, the regions potentially associated with gait apraxia were suggested by areas of reduced rCBF on SPECT. The two cases exhibited slightly different gait patterns, corresponding to functional impairments in different areas involved in the higher-order regulation of gait. In Case 1, reduced rCBF was observed in the dorsal SMA and pre-SMA, whereas in Case 2, the reduction was confined to the dorsal SMA. Moreover, diagnosing the rare symptom of gait apraxia in CBS can facilitate early diagnosis.

Gait apraxia lacks a clear, universal definition and is complicated by the fact that different authors have used various terms to describe walking disturbance, including gait apraxia. It is characterized by an inability to effectively use the lower limbs and trunk, causing a slowed, imbalanced gait with shortened steps, often appearing as “magnetic” [9]. Gait apraxia is distinguished from lower limb apraxia, which is assessed by testing conceptual movements such as kicking a ball or stubbing a cigarette with the foot [2]. Della Sala et al. [1] noted that, in contrast to ideomotor apraxia, gait apraxia affects routinized actions due to impairment of the system that controls sensorimotor and spatiotemporal movements, according to the dichotomy proposed by Benke [3]. We provisionally define “gait apraxia” as a higher-level gait disorder without lower limb apraxia and not attributable to other causes. The gait patterns observed in the two cases align with this definition and are suggestive of apraxic gait.

Higher-order regulation of gait is postulated to involve multiple brain regions, including the primary sensory cortex, basal ganglia, cerebellum, vestibular cortex, temporoparietal cortex, SMA, premotor cortex, and brain stem [6]. Focusing on cortical function rather than white matter connectivity, the SMA and premotor areas are particularly involved in somatosensation and motor programming. Furthermore, regarding the somatotopic organization of the SMA, the functional areas corresponding to the lower limb, upper limb, and face are distributed from dorsal to ventral regions [5]. Hence, impairment of the dorsal SMA could evoke lower limb dysfunction due to failure in higher-order regulation.

Freezing of gait may result from disruption of the basal ganglia and SMA loop responsible for initiating movement [7]. In terms of the distinction between pre-SMA and SMA, which are related to gait regulation, the pre-SMA is specifically activated during the planning and initiation of an action, whereas the SMA is activated during the movement execution stage [10]. Comparative analysis of brain imaging in patients with parkinsonism revealed that the pre-SMA is more disrupted in patients with freezing of gait [11]. Therefore, dysfunction of the pre-SMA may contribute to gait apraxia, which resembles a “freezing gait.”

In a case of stroke resulting from bilateral anterior cerebral artery infarction, gait apraxia with residual clumsiness after recovery from weakness is caused by damage to the first frontal gyrus, primarily affecting the SMA. Similarly, cases of central nervous system lymphoma and cerebral lobe atrophy exhibit apraxic gait, characterized by clumsiness and difficulty in initiating movement and making turns, owing to loss of function in the SMA. These findings suggest that blood flow reduction to a very limited area, specifically the SMA, predominantly induces apraxic gait rather than limb apraxia [12].

Both cases we described exhibit gait apraxia, and the symptoms were considered to be primarily related to the dysfunction of the SMA, as indicated by the reduction of rCBF on SPECT. Other areas, including the temporal and parietal lobes, also showed hypoperfusion, which may also have contributed to the gait apraxia. In Case 1, the walking pattern was characterized by consistent freezing and shuffling gait, whereas Case 2 exhibited peculiarities and clumsiness in the right foot during walking. These differences in gait patterns may be partly attributable to discrepancies in the impaired region of the SMA. The leg clumsiness in both cases is likely due to dysfunction of the dorsal SMA, which plays a role in controlling leg function according to the somatotopy. The characteristic gait pattern in Case 1, resembling freezing gait in the right leg, is probably due to a dysfunction in the pre-SMA, which is activated during the initiation phase of the movement. Although freezing gait can also be induced by dysfunction of the premotor area, there are no examination findings to support this in these two cases. Notably, CBS cases of truncal or lower limb apraxia onset have been reported; however, gait apraxia as an initial symptom is rare. A retrospective study revealed that 4 out of 24 patients manifested gait onset CBS, but no further details were provided [13]. Moreover, several leg-onset CBS cases diagnosed have been reported to date; however, cases of patients presenting with lower limb apraxia or gait apraxia as an initial symptom remain uncommon [14, 15]. Although gait apraxia is not included in the official CBS diagnostic criteria, it may indicate cerebral cortex dysfunction.

## Conclusion

Overall, in the present study, we reported two remarkable cases of CBS predominantly manifesting as slightly different gait apraxia, which appeared to be primarily associated with SMA dysfunction, as demonstrated by SPECT. Therefore, gait disturbances, even in the absence of weakness and limb apraxia, may be an early indicator of CBS.

## Supplementary Information

Supplementary Material 1: Video 1. First part: Video of the patient drawing circles and mimicking kicking a ball. The patient exhibited slight difficulties in performing the movements. Second part: Gait. The patient struggled with postural transitions, showing titubation and instability when rising from sitting. During walking, he pivoted on the left foot, with impaired movement of the right foot.

Supplementary Material 2: Video 2. First part: Video of the patient drawing circles and kicking a ball. The patient exhibited slight difficulties in performing the movements. Second part: Gait. During ambulation, the patient exhibited a peculiar gait pattern, lifting the right leg high, swinging it outward, and keeping the right ankle everted. He also walked with only the right foot scraping the ground, resembling a shuffling gait.

## Data Availability

No datasets were generated or analysed during the current study.

## Abbreviations

CBD: Corticobasal degeneration
CBS: Corticobasal syndrome
CSF: Cerebrospinal fluid
p-tau: Phosphorylated tau
rCBF: Regional cerebral blood flow
SMA: Supplementary motor area
t-tau: Total tau
